# 5 Year Outcomes of Patients With Aortic Structural Valve Deterioration Treated With Transcatheter Valve in Valve – A Single Center Prospective Registry

**DOI:** 10.3389/fcvm.2021.713341

**Published:** 2021-09-09

**Authors:** Nili Schamroth Pravda, Ran Kornowski, Amos Levi, Guy Witberg, Uri Landes, Leor Perl, Yaron Shapira, Katia Orvin, Raffael Mishaev, Yeela Talmor Barkan, Ashraf Hamdan, Ram Sharoni, Hana Vaknin Assa, Pablo Codner

**Affiliations:** ^1^Department of Cardiology, Rabin Medical Center, Petach Tikva, Israel; ^2^Sackler Faculty of Medicine, Tel Aviv University, Tel Aviv, Israel; ^3^Cardio-Thoracic Surgery Department, Rabin Medical Center, Petach Tikva, Israel; ^4^Affiliated to the Faculty of Medicine, Tel Aviv University, Tel Aviv, Israel

**Keywords:** ViV-TAVI, outcomes, structural valve deterioration, mortality, hemodynamic

## Abstract

The Valve-in-Valve (ViV) technique is an established alternative for the treatment of structural bioprosthetic valve deterioration (SVD). Data describing the intermediate term follow up of patients treated with this approach is scarce. We report on our intermediate-term outcomes of patients with SVD in the Aortic position treated with ViV. Included were patients with symptomatic SVD in the aortic position valve who were treated by Valve in valve transcatheter aortic valve implantation (ViV-TAVI) during the years 2010-2019 in our center. Three main outcomes were examined during the follow up period: NYHA functional class, ViV-TAVI hemodynamic per echocardiography, and mortality. Our cohort consisted of 85 patients (mean age 78.8 ± 8.9 years). The indications for aortic ViV were: SVD isolated aortic stenosis in 37.6%, SVD isolated aortic regurgitation in 42.2% and combined valve pathology in 20.0%. Self-expandable and balloon-expandable devices were used in 73 (85.9%) and 12 (14.1%), respectively. Average follow up was 3.7 ± 2.4 years. 95 and 91% of patients were in NYHA functional class I/II at 1 and 5 year follow up respectively. At one year, the mean trans-aortic valve pressure was 15 ± 9 mmHg and rates of ≥ moderate aortic regurgitation were 3.7%. Mortality at one year was 8.6% (95% CI 2.3–14.4) and 31% (95% CI 16.5–42.5) at 5 years. ViV in the aortic position offers an effective and durable treatment option for patient with SVD, with low rates of all-cause mortality, excellent hemodynamic and improved functional capacity at intermediate follow up.

## Introduction

Bioprosthetic surgical valve replacement for the treatment of native valve diseases is used extensively. The treatment of failed bioprosthetic valves has traditionally been open surgical valve replacement. However reoperation, especially in those at increased surgical risk, has associated substantial morbidity and mortality (1, 2). Valve in valve transcatheter aortic valve implantation (ViV-TAVI) inside failed surgically implanted bio-prostheses has become a reliable and less invasive alternative to repeat surgery (3, 4). We report herein on our clinical experience of treating patients with structural valve deterioration (SVD) in the aortic position using the ViV technique in our institution, aiming to provide insights into the clinical outcomes of these patients by providing intermediate-term follow up results.

## Materials and Methods

The characteristics and outcomes of patients with bioprosthetic SVD treated by the implantation of a Transcatheter aortic valve implantation (TAVI) device within a failed surgical valve, or valve-in-valve (ViV-TAVI) procedure, are described in the present report. The ViV-TAVI procedures were performed from November 2010 to December 2019. Patient data follow up was completed until December 2020. The selection and assessment process of these patients in our institution has previously been described (5). In addition to routine clinical assessment, from 2017 a gated cardiac CT and peripheral vessel scan was performed in all patients with detailed analysis of coronary height, sinus of Valsalva dimensions and virtual transcatheter heart valve to coronary ostial distance (VTC) assessment to assess the risk of coronary occlusion during the procedure. All patients undergo Transthoracic Echocardiograms and in selected cases also a transesophageal echocardiogram. Following an individualized analysis of patient biometrics parameters, bioprosthetic valve manufacturer characteristics and imaging characteristics, percutaneous valve device type and size were chosen on an individualized basis. Patients were treated empirically with dual antiplatelet therapy following the procedure. In patients with a prior indication for oral anticoagulation, the combination of oral anticoagulation with clopidogrel was given for 3–6 months following the procedure. In selected patients at high risk for bleeding, single antiplatelet with either aspirin or clopidogrel was used.

The baseline, procedural and peri-procedural findings are described. The prospective data collection was approved by the institutional review board. Three endpoints were examined: NYHA functional status at 1- and 5-year, valve hemodynamic competence of the implanted valves as per echocardiography done on an annual basis and rates of survival during the follow-up period. Data on mortality was based on mortality files derived from the notification of death form legally required by the Ministry of the Interior. Follow up data was available for 79 patients at one year follow up and 23 patients at 5 year follow up. Clinical events were defined according to the Valve Academic Research Consortium 2 (VARC 2) criteria (6). Structural valve deterioration (SVD) was defined as per consensus statement from the European Society of Cardiology of percutaneous cardiovascular interventions 2017 (7).

Baseline characteristics of the patients are presented as mean and standard deviation (SD) for continuous variables and count (%) for categorical variables. Continuous variables were compared using the Student's *t* test/Mann Whitney U test, categorical variables were compared using the chi-square/Fisher's exact test, as appropriate. All tests were 2 tailed, and a *p* value < 0.05 was considered significant. All-cause mortality was graphically plotted using Kaplan-Meier curves and compared between groups using the log rank test (unadjusted analysis). All TAVI-related data was registered in an electronic file and analyzed using the SPSS, version 25.0, software (SPSS, Chicago, Illinois).

## Results

Our cohort consisted of 85 patients in whom ViV in the aortic position was performed. Table 1 details the baseline characteristics of these patients. 45 (52.9%) were male and the mean age was 78.8 ± 8.9 years. This cohort had a mean STS score 6.1 ± 4.4 and most patients were in NYHA (New York Heart Association) functional class III/IV (78%) at baseline. The indication for ViV-TAVI were isolated xenograft stenosis in 37.6%, isolated xenograft regurgitation in 42.2% and combined pathology in 20.0%. 81 procedures were done for surgical xenografts and 4 procedures were done as TAVI in TAVI. The average time to ViV-TAVI from surgical valve intervention was 11.0 ± 1.3 years. The sizes and types of degenerated surgical valves and corresponding ViV-TAVI valves are detailed in Supplementary Table 1. Of the 85 patients included in our cohort 48 underwent a preprocedural gated cardiac CT scan. VTC was assessed before aortic ViV intervention in 42 (49.4%) patients, the mean VTC was 5.1 ± 1.4 mm. Table 2 details the Procedural Characteristics of patients who underwent ViV-TAVI. Vascular access was predominantly via the femoral route (91.8%). Self-expandable and balloon-expandable devices were used in 73 (85.9%) and 12 (14.1%), respectively. Major complications included: one periprocedural death due to a major vascular complication, one case of coronary artery occlusion, one ischemic stroke, one case of cardiac tamponade, 2 cases in which the ViV-TAVI migrated, and a second valve was needed. Other complications were rare: one case of stage 1 acute kidney injury and 2 minor vascular complications. There were 12 patients with new LBBB and 6 patients with new RBBB following the procedure. There were no cases in which a new permanent pacemaker was needed. There was a single case of moderate paravalvular leak after VIV-TAVI insertion. The average hospital stay was 4.8 ± 3.8 days.

**Table 1:** Baseline characteristics of the patients: valve-in-valve aortic position.

**Aortic**	***N* = 85**
Age (years)	78.8 ± 8.9
Male (%)	45 (52.9)
BMI	28.6 ± 5.7
STS	6.1 ± 4.4
Euroscore II	9.0 ± 5.8
Coronary artery disease (%)	47 (55.3)
Prior coronary artery bypass surgery (%)	37 (43.5)
Prior PCI (%)	24 (28.3)
Prior CVA/TIA (%)	9 (10.6)
Peripheral vascular disease (%)	8 (9.4)
Diabetes mellitus (%)	38 (44.7)
Hypertension (%)	79 (92.9)
Chronic obstructive pulmonary disease (%)	15 (17.6)
Atrial fibrilliation/flutter (%)	27 (31.7)
NYHA functional class III/IV (%)	65 (76.4)
Permanent pacemaker/defibrillator (%)	8 (9.4)
Hemoglobin (g/dL)	11.5 ± 1.7
GFR (MDRD)(mL/min/m^2^)	59.8 ± 24.1
Albumin (g/dL)	4.1 ± 0.4
Aortic valve peak pressure (mmHg)	56.1 ± 22.3
Aortic valve mean pressure (mmHg)	33.1 ± 14.5
Aortic valve area (cm^2^)	0.7 ± 0.2
Systolic pulmonary artery pressure (mmHg)	37.3 ± 19.2
Failed prosthesis size (mm)	
19	16 (19.8)
21	23 (28.4)
23	18 (22.2)
25 or above	28 (32.9)
Left ventricular systolic function	
Normal (>50%)	67 (78.8)
Mild (40–49%)	10 (11.7)
Moderate (30–39%)	5 (5.8)
Severe (<29%)	3 (3.5)
Valve Pathology	
Aortic Stenosis (%)	32 (37.6)
Aortic Regurgitation (%)	36 (42.4)
Combined (%)	17 (20.0)
Aortic Regurgitation (≥Moderate) (%)	40 (47.6)
Aortic Stenosis (≥Moderate) (%)	49 (57.6)

*BMI, Body mass index; STS, Society of Thoracic Surgeons; PCI, Percutaneous Coronary Intervention; CVA/TIA, Cerebrovasclar Accident/Transient Ischemic Attack; GFR, glomerular Filtration rate; MDRD, Modification of Diet in Renal Disease*.

**Table 2:** Procedural Characteristics: valve-in-valve aortic position.

Fluoroscopy time (min)	22.3 ± 11.2
Contrast volume (ml)	112.6 ± 49.6
Procedure urgent (%)	16 (18.8)
Anesthesia	
Conscious sedation or local anesthesia only (%)	55 (64.7)
General Anesthesia (%)	30 (35.3)
Vascular Access (%)	
Femoral	78 (91.8)
Apical	1 (1.2)
Axillary	5 (5.9)
Surgical cut down	1 (1.2)
Concomitant PCI (%)	8 (9.4)
Self-expandable valve (%)	73 (85.9)
Balloon expandable valve	12 (14.1)
Balloon post-dilation	34 (40.0)
ViV-TAVI size (mm)	
23	41 (48.2)
26	32 (37.6)
29	11 (12.9)
34	1 (1.2)

*PCI, Percutaneous Coronary Intervention; ViV-TAVI, Valve in valve transcatheter aortic valve implantation*.

**Figure 1:**
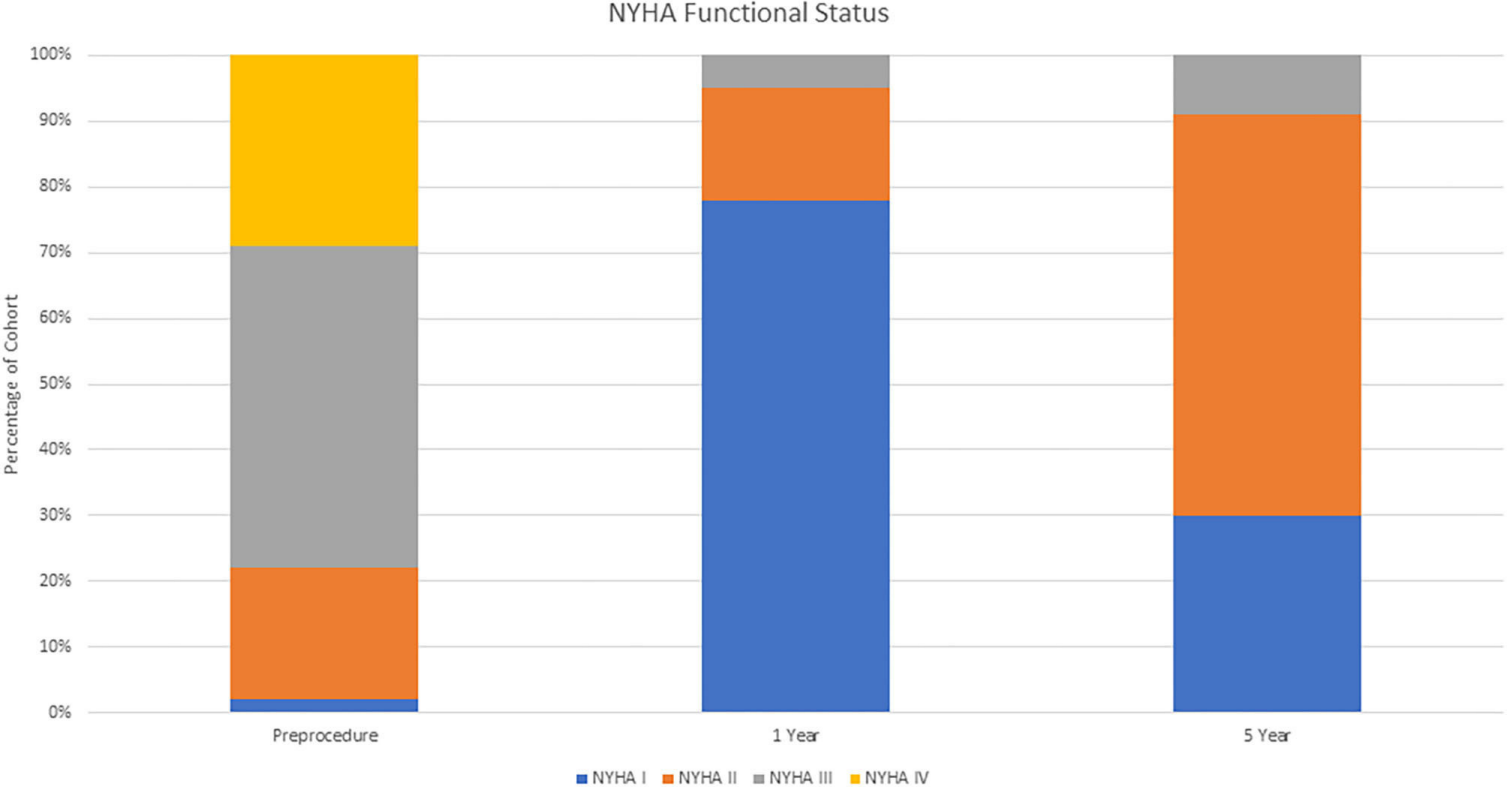
NYHA functional class over time.

**Figure 2:**
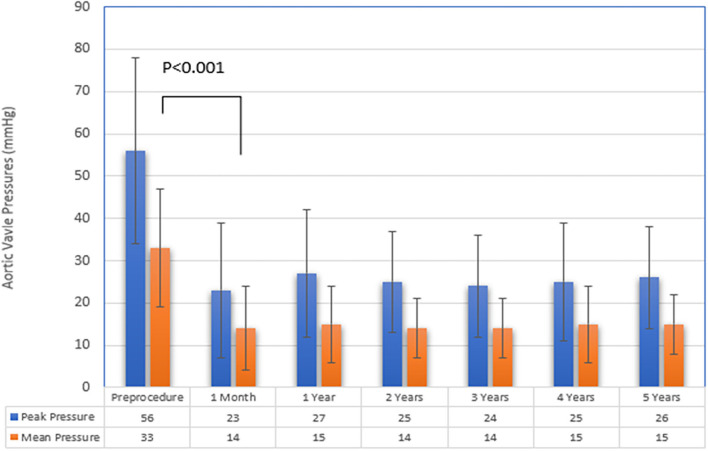
Aortic Valve Pressures during follow up.

Average time of follow up was 3.7 ± 2.4 years. Most patients were in NYHA functional status I/II at 1 and 5 year follow up (95 and 91%, respectively) (Figure 1). There were significantly more patients in NYHA I/II vs NYHA III/IV at one year than at baseline (*p* = 0.04). This improvement from baseline was seen at 5 years in the 23 patients available for comparison, but was not statistically significant (*p* = 0.08). A favorable hemodynamic valve profile was maintained over the follow up as seen in Figure 2. At 1-month follow up, the valve gradients were significantly reduced from mean pressure of 33 ± 14 mmHg to mean 14 ± 10 mmHg (*p* < 0.001). This reduction in valve gradients were maintained over time. 1-year average mean transaortic valve gradient was 15 ± 9mmHg, with low rates of ≥ moderate aortic regurgitation (3.7%) (Supplementary Figure 1). At 1 year follow up, patients with larger deteriorated bioprosthetic valves (larger than 21 mm) had significantly lower mean valve gradients compared to those implanted with smaller valves ( ≤ 21 mm) (14 ± 7 mmHg vs. 18 ± 11 mmHg, *p* = 0.04; respectively) (Table 3). There were no differences in mortality rates between patients with smaller in comparison to larger deteriorated bioprosthetic valves during the follow up period.

**Table 3:** Aortic Valve pressures comparing large and small valves.

	**Large valve (>21 mm)**	**Small valves (≤21 mm)**	***P* value**
Aortic Valve mean Pressure at baseline (mmHg)	30.8 ± 14.7	34.4 ± 12.8	0.299
Aortic Valve mean Pressure at 1 Year (mmHg)	13.8 ± 7.6	18.5 ± 10.9	0.04

**Figure 3:**
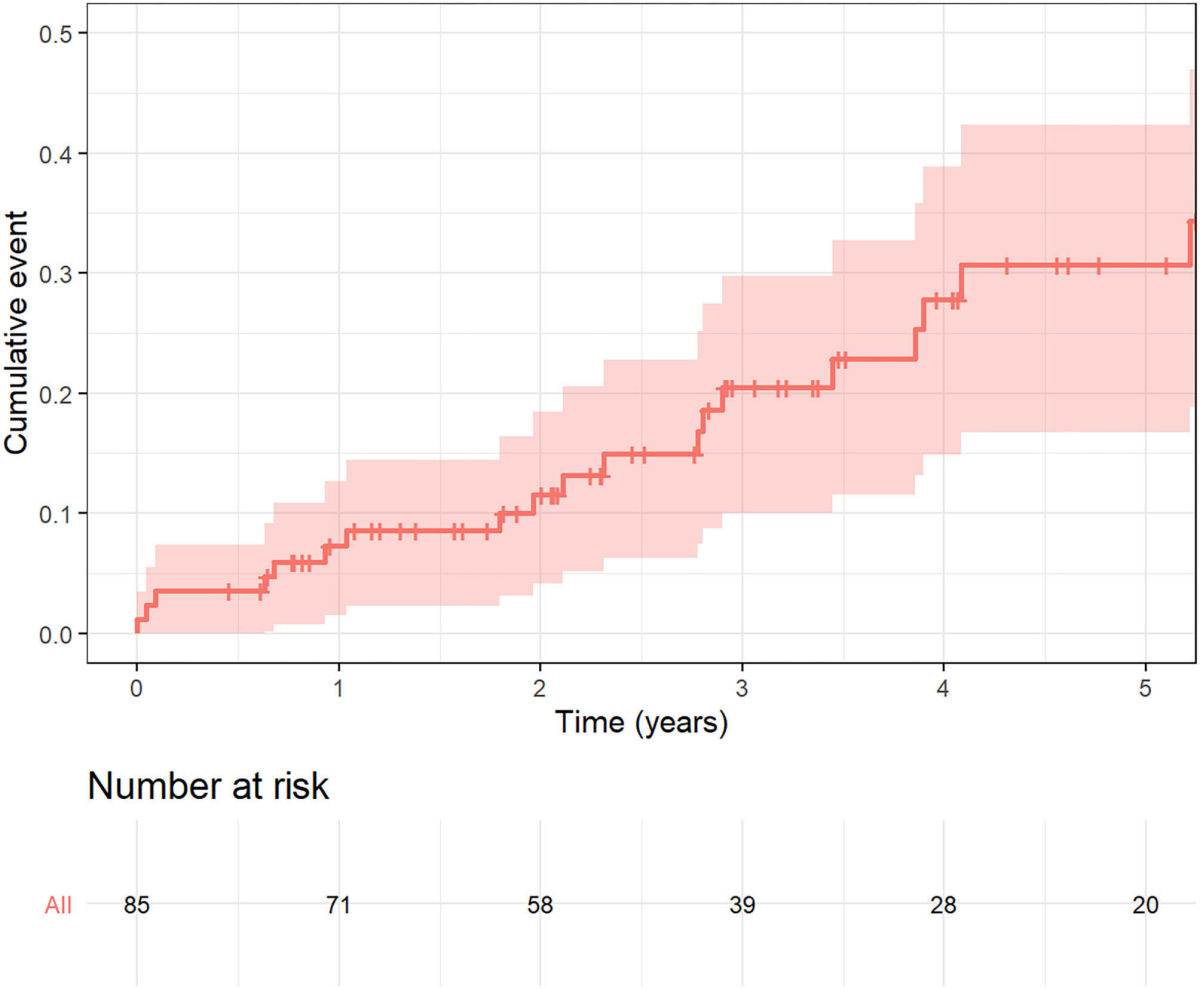
Kaplan Meier curve for mortality during follow up.

Kaplan Meier mortality rates at one year was 8.6% (95% CI 2.3–14.4) and 31% (95% CI 16.5–42.5) at 5 years (Figure 3). Of the 22 patients who died during follow up, only 3 patients died from cardiovascular causes (13.6 %). There was a single peri-procedural death due to a major vascular complication.

## Discussion

The main objective of our study was to report on the intermediate-term clinical outcomes of patients with SVD treated with ViV-TAVI from our all-comer perspective registry. The main findings are as follows: Firstly, patients in our cohort had a significant improvement in functional status following TAVI-ViV at 1 and 5 year follow up. Secondly, the immediate achieved trans-valvular gradients were favorable and maintained over the duration of the follow-up period. Thirdly, all-cause mortality at 1 year and 5 year follow up was low, with mortality rate at one year of 8.6% (95% CI 2.3–14.4) and 31% (95% CI 16.5–42.5) at 5 years.

Our findings correlate with other reports reporting a significant improvement in functional status following ViV-TAVI (4, 8). Dvir et al. reported that 92.6% of surviving patients were at NYHA I/II at one month following ViV-TAVI. We confirm and have expanded on this and found that 95% of surviving patients in our cohort had NYHA I/II at 1 year follow up. Moreover, this improvement in functional status was maintained at 5 year follow up with 91% of patients being in NYHA I/II. The ViV-TAVI procedure provided a meaningful and persistent improvement in quality of life. Valve hemodynamics in our analysis are in concordance with previous reports, at 1-year the average mean aortic pressure was 15.3 ± 9.5 mmHg (8, 9). These favorable hemodynamics were maintained during follow up period. One of the challenges in ViV-TAVI is that there is an element of patient prothesis mismatch (PPM) that cannot be discounted due to the reduced potential orifice area for ViV-TAVI within the dimension of the failed bioprosthetic surgical valve. In a meta-analysis by Head et al. up to 44.2% of patients undergoing aortic valve replacement have some degree of PPM following surgery (10). This is even more pronounced in patients with small bioprosthetic degenerated valves ( ≤ 21mm) and has been associated with poorer long term outcomes (8). In our experience the mean postprocedural transaortic gradient at 1 year follow up was significantly higher in those with smaller deteriorated bioprosthetic valves ( ≤ 21 mm) compared to those with larger deteriorated bioprosthetic valves (>21 mm) (18 ± 11 mmHg vs. 14 ± 7 mmHg, *p* = 0.04). This did not reflect a survival disadvantage, although this is confounded by our relatively small sample size. Dvir et al. reported that the rate of elevated postprocedural gradients were significantly higher after balloon expandable than after self-expandable in patients after aortic ViV interventions (40 vs. 21%; *P* < 0.0001) (11). In order to minimize PPM, the majority of cases in our institution for ViV-TAVI are performed with the CoreValve Evolut system with high rates of post dilation balloon (40%). This valve device has a supra-annular feature and so the component with the functioning valve is less affected by the surgical bioprosthesis dimensions, enabling for a larger effective orifice area. Bioprosthetic valve fracture has been reported as another safe and effective alternative to reduce post procedural gradients in ViV-TAVI procedure (12). However this technique has yet to be evaluated in a large randomized control trial and is not routinely done in our institution.

All-cause mortality rates in our cohort were low and most mortality events were secondary to non-cardiac causes (86.4%).

One would expect that the mortality in our cohort would be less than that reported in patients undergoing native valve TAVI. However, our reported mortality is lower than that reported in registries of patients undergoing native TAVI. Other registries such as GARY (German Aortic valve registry) in patients with intermediate risk reported a 17.5% one-year mortality in those undergoing TAVI and 10.8% one-year mortality in those undergoing surgical aortic valve replacement (AVR) (13). The PARTNER 2 investigators reported on 5 year results in those with intermediate risk with a reported mortality of 46% in those with TAVI and 42.1% in this with surgical AVR (14). Mortality rates in our cohort at one year was 8.6% (95% CI 2.3–14.4) and 31% (95% CI 16.5–42.5) at 5 years. The discrepancy between these findings is most likely due to the inherent differences in patients undergoing native TAVI and ViV-TAVI. Although these patients have had previous surgery, those selected for the procedure are those that have a clinical state deemed fit to undergo the procedure with expected longevity. There is a selection bias of the cohort. Furthermore, the weight of previous cardiac surgery in the calculation of surgical risk in these patients may be over-estimated. Our institution has a meticulous patient selection process, a well-established institutional heart team, and an increased experience of operators and post procedural care.

The procedural complications reported in our cohort were low. The dreaded Achilles' heel of ViV-TAVI is coronary obstruction (9). Coronary obstruction is primarily caused by coronary ostium occlusion by the displaced leaflet of the bioprosthetic valve following deployment of the ViV-TAVI. Computer tomography (CT) imaging plays a central role in identifying high-risk features for coronary obstruction such as short virtual transcatheter heart valve to coronary ostial distance (VTC) <4 mm (15). Pre-procedural CT has been performed routinely in all ViV-TAVI cases in our institution since mid-2016. VTC in combination with other parameters (type of deteriorated surgical valve: stented vs. stent-less, with or without externally mounted leaflets, coronary height) were assessed for procedural planning. In selected cases, procedures were performed with “coronary” wire protection and stent implantation (chimney technique), or a repositionable/recapturable transcatheter valve was used specifically to avoid coronary occlusion. Novel transcatheter techniques such as Basilica may become increasingly used in high risk patients (16). There were no cases of new permanent pacemaker insertion following the procedure. There is data to show that the rate of need for new permanent pacemaker insertion following ViV-TAVI is far less that that seen in native TAVI. This could be due to the relative protection of the conduction system by the surgical valve structure from the mechanical pressure of the ViV-TAVI device (17).

The main strengths of our study are the quality of our data acquisition and the intermediate-term follow up. We are a center with a dedicated structural invention team with increasing experience and data spanning over 10 years. We have a dedicated data collection team and structured clinical and imaging follow up program to ensure careful data acquisition and quality. Study limitations include the single-center nature of this observational study. Our echo data did not routinely include aortic valve area on follow up and thus we did not include data on patient-prosthetic mismatch on follow up. The ViV-TAVI procedure offers a transcatheter solution to a heterogenous group of bioprosthetic valve dysfunction. Due to the heterogeneity in the type and size of bioprosthetic valves and small size of our cohort, we did not do further subgroup analysis. With increasing follow up data on ViV-TAVI procedures, there is a need for dedicated standardized criteria for ViV-TAVI procedures. VARC 2 criteria were derived for TAVI in native aortic valves. However, the ViV-TAVI procedure has unique features and challenges for which VARC 2 criteria are inadequate. One of the prominent features is the PPM at baseline, and VARC2 criteria are misrepresentative of hemodynamic SVD in ViV-TAVI.

While the ViV-TAVI procedure requires operator experience and adequate preprocedural planning, this procedure offers a less invasive, safe solution to bioprosthetic valve deterioration. Our real-world single center data shows promising intermediate-term results of patients undergoing ViV-TAVI. Our results of ViV-TAVI for the treatment of symptomatic bioprosthetic valve failure yielded encouraging results in terms of clinical efficacy, durability and hemodynamic profile of ViV-TAVI.

## Data Availability Statement

The raw data supporting the conclusions of this article will be made available by the authors, without undue reservation.

## Ethics Statement

The studies involving human participants were reviewed and approved by Helsinki Ethics Committee Rabin medical Center. Written informed consent for participation was not required for this study in accordance with the national legislation and the institutional requirements.

## Author Contributions

All co-authors contributed substantial contributions to the conception or design of the work, or the acquisition, analysis or interpretation of data for the work, including drafting the work or revising it critically for important intellectual content, provide approval for publication of the content, and agree to be accountable for all aspects of the work in ensuring that questions related to the accuracy or integrity of any part of the work are appropriately investigated and resolved.

## Conflict of Interest

The authors declare that the research was conducted in the absence of any commercial or financial relationships that could be construed as a potential conflict of interest.

## Publisher's Note

All claims expressed in this article are solely those of the authors and do not necessarily represent those of their affiliated organizations, or those of the publisher, the editors and the reviewers. Any product that may be evaluated in this article, or claim that may be made by its manufacturer, is not guaranteed or endorsed by the publisher.
