# Supplementary material for: 5 Year Outcomes of Patients With Aortic Structural Valve Deterioration Treated With Transcatheter Valve in Valve – A Single Center Prospective Registry
Source: Front Cardiovasc Med. 2021 Sep 9;8:713341. doi: 10.3389/fcvm.2021.713341 (PMC8458695; doi:10.3389/fcvm.2021.713341)
Supplement: Supplementary file 1 [file Table_1.DOCX]

Table S1: Bioprosthetic Valve Types and ViV Valve Types Detailed

| **Degenerative Aortic valve type** | **Bioprosthetic Aortic valve size (mm)** | **ViV Valve type** | **VinV valve size (mm)** | **Time from bioprosthetic surgical valve to VinV (Years)** |
| --- | --- | --- | --- | --- |
| Centera (Edwards) | 21 | Edwards SAPIEN 3 | 23 | 12 |
| Centera (Edwards) | 25 | Medtronic Evolute PRO | 26 | 14 |
| Edwards SAPIEN 3 | 23 | Evolut R | 23 | 6 |
| Evolute PRO | 25 | Edwards SAPIEN 3 | 26 | 9 |
| Freedom Solo | 25 | Medtronic Evolute R | 29 | 12 |
| Freestyle | 29 | Medtronic Evolute R | 34 | 10 |
| Freestyle | 27 | Medtronic Evolute R | 29 | 16 |
| Freestyle Valve | 21 | SYMETIS | 23 | 7 |
| Hancock | 27 | Edwards SAPIEN / XT | 26 | 10 |
| Hancock | 25 | Edwards SAPIEN 3 | 23 | 14 |
| Hancock | 23 | Medtronic Evolute R | 26 | 12 |
| Homograft | 23 | Edwards SAPIEN 3 | 29 | 17 |
| Homograft | 23 | Medtronic Evolute R | 29 | 13 |
| Homograft | 23 | Edwards SAPIEN 3 | 29 | 19 |
| Magna | 25 | Medtronic Evolute R | 26 | 7 |
| Magna | 23 | Medtronic Evolute R | 23 | 8 |
| Magna | 21 | Medtronic Evolute R | 23 | 12 |
| Magna | 19 | Medtronic Evolute R | 23 | 9 |
| Magna | 21 | Medtronic Evolute R | 23 | 7 |
| Magna | 19 | Medtronic Evolute PRO | 23 | 7 |
| Magna | 21 | Medtronic Evolute PRO | 23 | 9 |
| Magna | 21 | Medtronic Evolute PRO | 26 | 4 |
| Magna | 25 | Medtronic Evolute PRO | 29 | 12 |
| Magna | 23 | Medtronic Corevalve | 26 | 6 |
| Mitoflow | 19 | Medtronic Corevalve | 23 | 9 |
| Mitroflow | 21 | Medtronic Corevalve | 26 | 18 |
| Mitroflow | 19 | Medtronic Corevalve | 23 | 10 |
| Mitroflow | 19 | Medtronic Evolute R | 23 | 8 |
| Mitroflow | 21 | Medtronic Evolute R | 23 | 12 |
| Mitroflow | 19 | Medtronic Evolute R | 23 | 11 |
| Mitroflow | 21 | Medtronic Evolute PRO | 23 | 9 |
| Mitroflow | 19 | Medtronic Evolute PRO | 23 | 11 |
| Mitroflow | 21 | Medtronic Corevalve | 26 | 14 |
| Mitroflow | 21 | Medtronic Corevalve | 26 | 21 |
| Mitroflow | 23 | Medtronic Corevalve | 26 | 7 |
| Mitroflow | 19 | Medtronic Corevalve | 23 | 9 |
| Mitroflow | 19 | Medtronic Corevalve | 23 | 9 |
| Mitroflow | 19 | Medtronic Corevalve | 23 | 6 |
| Mitroflow | 21 | Medtronic Corevalve | 23 | 5 |
| Mitroflow | 19 | Medtronic Corevalve | 23 | 15 |
| Mitroflow | 19 | Medtronic Corevalve | 23 | 11 |
| Mitroflow | 19 | Medtronic Corevalve | 23 | 4 |
| Mitroflow | 21 | Medtronic Corevalve | 23 | 11 |
| Mitroflow | 19 | Medtronic Evolute R | 23 | 9 |
| Mitroflow | 21 | Medtronic Evolute R | 23 | 11 |
| Mitroflow | 25 | Medtronic Evolute R | 26 | 8 |
| Mosaic | 23 | Medtronic Corevalve | 26 | 11 |
| Mosaic | 23 | Medtronic Corevalve | 26 | 16 |
| Mosaic | 25 | Medtronic Evolute R | 26 | 6 |
| Mosaic | 23 | Medtronic Evolute R | 23 | 12 |
| Mosaic | 27 | Medtronic Evolute PRO | 29 | 13 |
| Mosaic | 25 | Edwards SAPIEN 3 | 26 | 15 |
| Mosaic | 25 | Medtronic Corevalve | 26 | 10 |
| Perceval | 21 | Edwards SAPIEN / XT | 23 | 3 |
| Perceval |  | Edwards SAPIEN 3 | 23 | 1 |
| Perimount | 23 | Medtronic Corevalve | 26 | 13 |
| Perimount | 23 | Medtronic Corevalve | 23 | 2 |
| Perimount | 21 | Medtronic Corevalve | 26 | 6 |
| Perimount | 23 | Medtronic Evolute R | 26 | 17 |
| Perimount | 21 | Medtronic Evolute R | 23 | 15 |
| Perimount | 21 | Medtronic Evolute R | 23 | 17 |
| Perimount | 23 | Medtronic Evolute R | 26 | 14 |
| Perimount | 23 | Medtronic Evolute R | 26 | 13 |
| Perimount | 21 | Medtronic Evolute R | 23 | 11 |
| Perimount | 25 | Edwards SAPIEN 3 | 26 | 27 |
| Perimount |  | Medtronic Evolute PRO | 23 | 10 |
| Perimount | 25 | Edwards SAPIEN 3 | 26 | 10 |
| Perimount | 27 | Medtronic Evolute R | 29 | 6 |
| Perimount | 19 | Medtronic Evolute R | 23 | 11 |
| Perimount | 25 | Medtronic Evolute R | 26 | 4 |
| Perimount | 25 | Edwards SAPIEN 3 | 26 | 8 |
| Perimount | 21 | Medtronic Evolute PRO | 23 | 19 |
| Perimount | 19 | Medtronic Evolute PRO | 23 | 6 |
| Perimount | 25 | Medtronic Evolute PRO | 26 | 11 |
| Perimount | 21 | Medtronic Evolute PRO | 23 | 10 |
| Perimount | 25 | Medtronic Evolute PRO | 26 | 12 |
| Perimount | 21 | Medtronic Evolute PRO | 23 | 12 |
| Perimount | 25 | Medtronic Evolute PRO | 29 | 9 |
| Perimount | 25 | Medtronic Evolute R | 26 | 12 |
| Toronoto SPV | 29 | Medtronic Corevalve | 29 | 14 |
| Toronto SPV | 23 | Medtronic Corevalve | 26 | 16 |
| Toronto SPV | 25 | Medtronic Corevalve | 26 | 16 |
| Xenograft | 25 | Medtronic Evolute R | 23 | 16 |
| Xenograft | 23 | Medtronic Evolute PRO | 29 | 12 |
