# Supplementary figures and images for: 5 Year Outcomes of Patients With Aortic Structural Valve Deterioration Treated With Transcatheter Valve in Valve – A Single Center Prospective Registry
Source: Front Cardiovasc Med. 2021 Sep 9;8:713341. doi: 10.3389/fcvm.2021.713341 (PMC8458695; doi:10.3389/fcvm.2021.713341)

## Slide 1
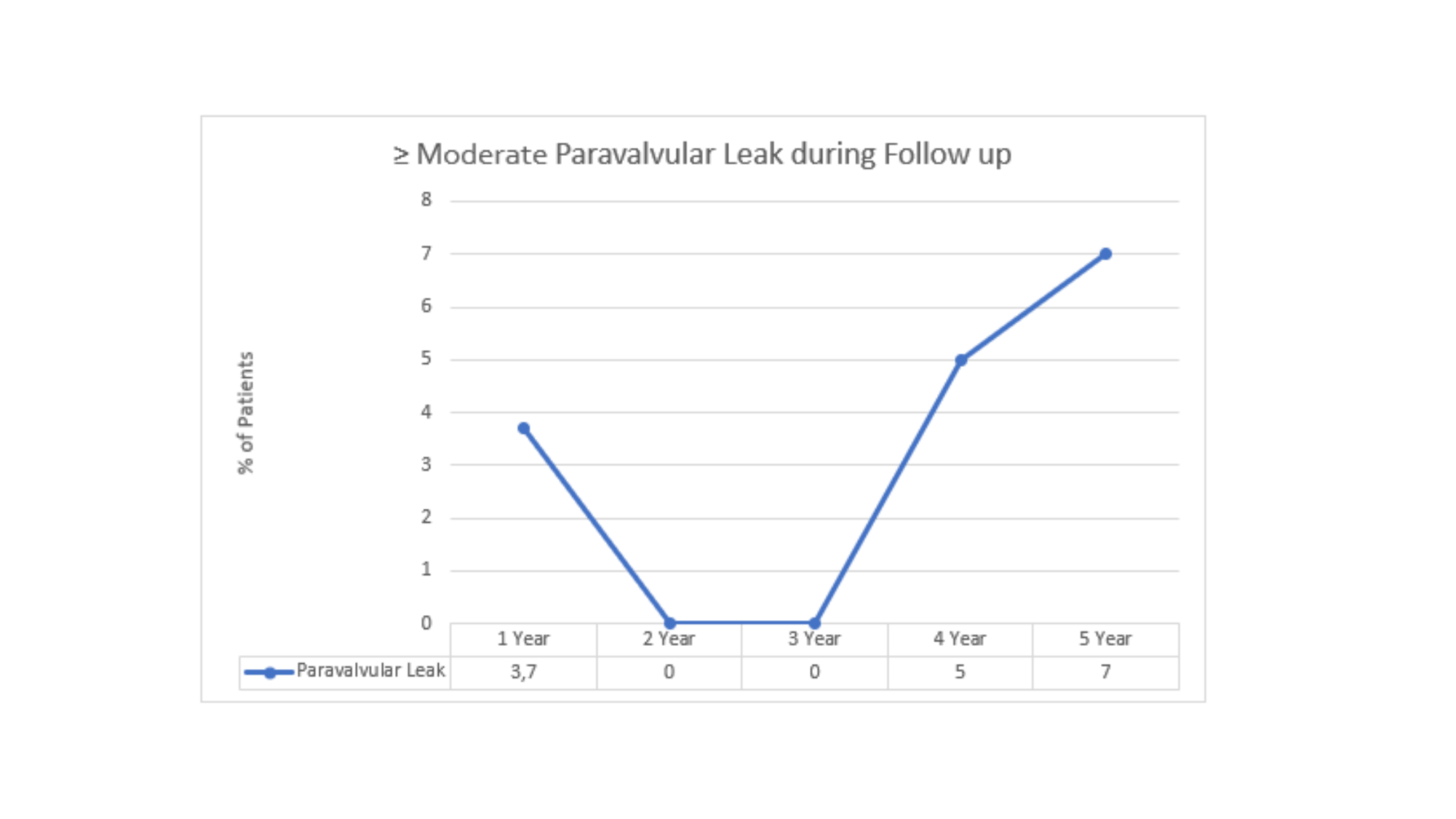

Supplement: Supplementary file 2 [file Presentation_1.PPTX]
